# Single-Incision Direct Lateral Approach Versus Dual-Incision Approach for Distal Tibial and Fibular Fractures

**DOI:** 10.7759/cureus.69516

**Published:** 2024-09-16

**Authors:** Jitendra Mishra, Tapan Kumar Das, Kshitij Guglani, Sudarsan Behera, Nego Zion, Prasanna Biradar

**Affiliations:** 1 Department of Orthopaedics, Institute of Medical Sciences and SUM Hospital, Bhubaneswar, IND; 2 Department of Orthopaedics, All India Institute of Medical Sciences, Deoghar, IND

**Keywords:** anterolateral distal tibia plate, delayed wound healing, direct lateral approach, distal tibia and fibula fractures, distal tibia fracture, distal tibia locking compression plate, mipo tibia, orif distal tibia fibula, pilon fractures, single-incision plating

## Abstract

Introduction: Distal tibial and fibular fractures are typically the result of high-energy trauma. Open reduction and internal fixation (ORIF) are often used to reconstruct and reduce displaced fractures, especially intra-articular ones. These fractures can be addressed either by a dual-incision approach (medial approach for the distal tibia and lateral approach for the fibula) or by a single-incision direct lateral approach to fix both the tibia and fibula. The direct lateral approach avoids injury to the medial soft tissues. This study was conducted to compare the postoperative clinico-radiological and functional outcomes of the single-incision direct lateral approach and the dual-incision approach for distal tibial and fibular fractures.

Materials and methods: A prospective comparative cohort study of 40 patients was conducted. The patients were classified into two cohorts of 20 each based on the surgical approach: those who underwent a single-incision direct lateral approach and those who underwent a dual-incision approach for distal tibial and fibular fractures (procedure: ORIF with plating). The study was conducted from September 2022 to March 2024. A follow-up period of at least 12 months was carried out, comparing operative time, discharge time, and postoperative outcomes using the American Orthopaedic Foot and Ankle Society (AOFAS) ankle-hindfoot score, ankle range of motion (ROM), Southampton wound score for wound healing, visual analog scale (VAS) pain score, and periodic radiographs at each follow-up. Complications were also studied.

Results: The mean operative time was 95.06 ± 7.04 minutes for the single-incision approach and 109.89 ± 7.88 minutes for the dual-incision approach. The average blood loss was 202.41 ± 32.76 mL for the single-incision approach and 248.39 ± 28.18 mL for the dual-incision approach. The hospital stay was shorter in the direct lateral approach group, and the AOFAS score at 12 months was better in the direct lateral approach group (91.47 ± 2.55 for the single-incision approach vs. 83.33 ± 8.71 for the dual-incision approach). Postoperative wound healing was observed, and the Southampton wound score was compared. Overall, soft tissue complications were fewer in the direct lateral approach group. The postoperative VAS pain score was consistently lower in the single-incision direct lateral approach group, which also demonstrated better ankle ROM. The p-value was significant (<0.05) for these parameters. At the six-month follow-up, all patients exhibited clinical and radiographic healing and bone union, except for one case in the dual-incision group. A medial compound wound, treated by plastic surgery with flap cover intervention, was identified as one of the definitive indications for single-incision plating.

Conclusion: The single-incision approach was associated with better soft tissue healing, fewer wound complications, and superior ankle functional outcomes compared to the dual-incision approach.

## Introduction

Distal tibial fractures account for 7.5% of all tibial fractures, and 75% of the time, they are associated with distal fibular fractures. These fractures are challenging injuries to treat. Open reduction and internal fixation (ORIF) is often used to reconstruct and reduce displaced fractures, particularly intra-articular fractures.

These fractures are typically caused by high-energy trauma, such as falls from heights resulting in axial compressive injuries, or by direct bending or rotational forces from motor vehicle accidents. Soft tissue complications and delayed or non-union are common outcomes of distal tibial and fibular fractures due to their origin, fracture patterns, and anatomical features [1].

Nowadays, fibular fractures are typically fixed first, with the ORIF of distal tibial fractures delayed. In most cases, temporary spanning external fixation of the medial side of the ankle, combined with immediate ORIF of the fibula, is employed [2]. The risk of serious wound complications with early ORIF of the distal tibia, whether via anteromedial or anterolateral approaches, is the primary reason for delaying ORIF [3]. The anteromedial incision allows for the insertion of a medial buttress plate to stabilize the comminuted metaphyseal region of the fracture while also providing good exposure to the tibial articular surface, both medially and centrally [4]. However, it is less advantageous for addressing the syndesmosis and the lateral articular surface of the distal tibia, which is particularly crucial when there is a lateral fragment of the tibial lateral plafond detached from the fibula [5].

When complications arise from the anteromedial/medial approach, the implant in the distal tibia may become exposed. The subcutaneous placement of an anteromedial plate may cause discomfort to patients, even when healing proceeds without incident [6]. Surgical site complications associated with a dual-incision approach, due to trauma to the fragile medial soft tissues, are a significant concern. Some studies have recommended minimally invasive percutaneous plating via a medial approach; however, the skin overlying the medial malleolus, where the plate is inserted, is often thin and frequently damaged in cases of displaced and/or comminuted fractures [7].

The anterolateral approach for treating distal tibial pilon fractures has been described in detail [8]. This method involves making a skin incision along the anterior border of the fibula, between the distal tibia and fibula. By using this approach, the delicate medial soft tissues are preserved, and only one incision is required to plate both the fibular and tibial fractures. The single-incision approach also provides good visualization of the distal articular surface and lateral column of the tibia [9]. However, the superficial peroneal sensory nerve is at risk with this method and must be identified and protected.

According to the angiosome concepts outlined by Taylor et al. in 1988 [10], the leg can be divided based on the skin's blood supply to better understand the surgical anatomy, particularly in the distal leg and ankle. In the dual-incision method, the anteromedial incision disrupts the perforators of the anterior tibial artery, while the lateral incision along the posterior border of the fibular disrupts the perforators of the peroneal artery. As a result, wound dehiscence is more likely to occur over the tibial and fibular hardware.

Clemens et al. (2010) used the angiosome concept and stated that, for better wound healing, incisions should be placed between angiosomes. In distal tibial and fibular reconstruction, their study showed that a single anterolateral incision to the distal tibia and fibula could preserve the angiosomes and prevent postoperative wound necrosis [11].

When ORIF with dual incisions is chosen as the management for fractures, two distinct incisions are made. If the combined incisions are separated by a distance of 7 cm or less, a poor outcome may be inevitable due to excessive soft tissue injury and periosteal damage [12].

Fractures of the distal tibia and fibula that are metaphyseal and extra-articular or partial articular can be approached by both the direct lateral single-incision approach and the dual-incision approach. When there is a major medial intra-articular fracture fragment, the dual-incision approach is recommended.

To overcome the limitations of the dual-incision approach, a single-incision approach has been suggested for the treatment of distal tibial and fibular fractures. Research on the clinical and functional outcomes of this approach, including rates of skin slough, infection, union, and ankle range of motion (ROM), remains limited. Our study is based on the hypothesis that the single-incision approach offers better clinical and functional outcomes, particularly in terms of soft tissue preservation and wound healing compared to the dual-incision approach.

## Materials and methods

This is a prospective comparative cohort study conducted at the Department of Orthopedics, Institute of Medical Sciences and SUM Hospital (IMS and SUM Hospital), Bhubaneswar, over a period of one year and six months (from September 2022 to March 2024). The Institutional Ethics Committee approved the study (approval number: IEC/IMS.SH/SOA/2023/699). Patients with a diagnosis of closed distal tibial and fibular fractures, presenting to the ER or OPD and admitted to IMS and SUM Hospital, were included in the study after obtaining their informed written consent. Adult patients over the age of 18 years with extra-articular, partial, or intra-articular distal tibial and fibular fractures were included. Old distal tibial and fibular fractures that were initially open but became closed after appropriate wound management were also included. Diaphyseal fractures were excluded, as were patients with unsuitable skin conditions of the ankle for surgery due to open fractures requiring external fixation or as a result of systemic diseases such as uncontrolled diabetic microangiopathy, neuropathy and vasculopathy, peripheral vascular disease due to atherosclerosis, Buerger’s disease, varicose veins, or lipodermatosclerosis. The patients were divided into two groups of 20 patients each: single-incision direct lateral approach and dual-incision approach (minimally invasive plate osteosynthesis (MIPO)/ORIF tibia + ORIF fibula). The assignment to a single- or dual-incision approach was made based on the surgeon's choice, depending on the fracture pattern and preoperative planning.

Single-incision direct lateral approach

**Figure 1:**
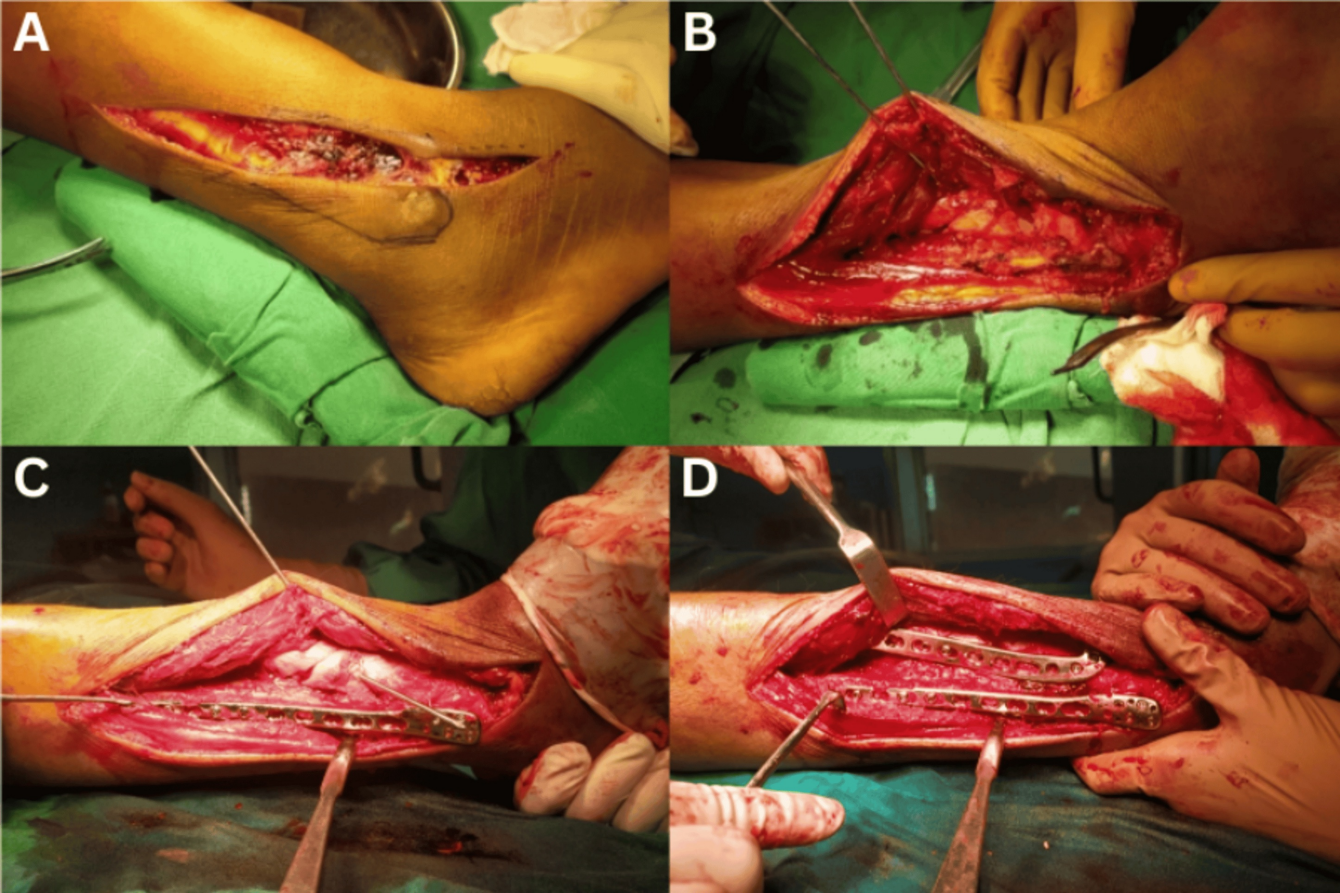
Intraopertive images showing the steps of single-incision direct lateral approach to the distal tibia and fibula (A) The incision was made on the skin and subcutaneous tissues along the anterior border of the fibula, between the tibia and fibula. The distal end of the incision was aligned with the fourth ray. (B) The peroneus longus and brevis muscles were retracted laterally and posteriorly, while the peroneus tertius and extensor digitorum were retracted medially and anteriorly. (C) The fibula was exposed and fixed first. (D) After the reduction of the tibial fracture was achieved, an anterolateral L-anatomical locking plate was applied to the lateral aspect of the tibia for fixation.

Along the anterior edge of the fibula, a longitudinal incision was made in the skin and subcutaneous tissues between the tibia and fibula (following the direction of the extensor digitorum longus muscle). The incision's distal end was aligned with the fourth ray (Figure 1). Depending on the need for internal fixation along the anterior fibular boundary, the proximal end of the incision may need to be extended further proximally. First, the fibular fracture was exposed, and the fibularis longus and brevis muscles were retracted posteriorly. The fracture was fixed with either a one-third tubular plate or a distal fibular anatomical locking plate, depending on the location of the fracture line. The extensor digitorum longus and superficial peroneal nerve should be carefully retracted medially. In the proximal portion of the fracture, the extensor digitorum longus, extensor hallucis longus, and tibialis anterior muscles were all retracted medially. To reveal the distal tibia, the tibialis anterior and deep peroneal nerve were withdrawn medially at the distal end. An anterolateral L-anatomical locking plate was placed on the lateral portion of the tibia for fixation after the tibial fracture had been reduced. An allogenic bone graft was used in cases where excessive comminution or a metaphyseal bone gap was present, sourced from the contralateral iliac crest.

Dual-incision approach

**Figure 2:**
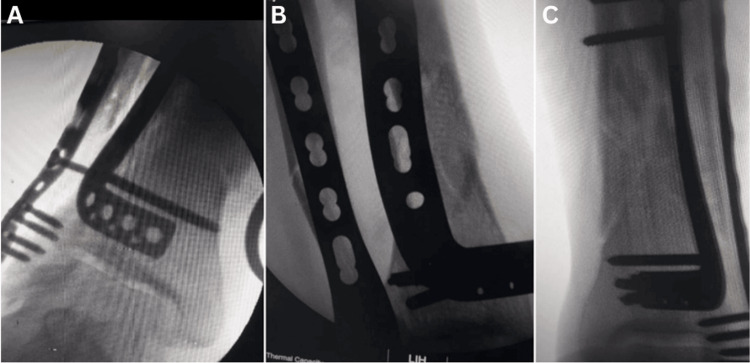
Intraoperative fluoroscopic images of the case as shown in Figure 1

For the lateral fibular approach, make a 10- to 15-cm incision along the fibula, centered above the fracture (Figure 2). The dissection plane is located between the peroneus longus and brevis posteriorly and the anterior peroneus tertius. The superficial peroneal nerve is located quite near the anterior aspect; take great care not to injure it, particularly in the proximal region of the incision. It needs to be recognized and protected in more anterior incisions. Be cautious not to harm the sural nerve or the short saphenous vein when dissecting posteriorly. Perform direct fracture reduction and fixation with a one-third fibular plate or anatomical distal fibular plate, followed by routine closure.

**Figure 3:**
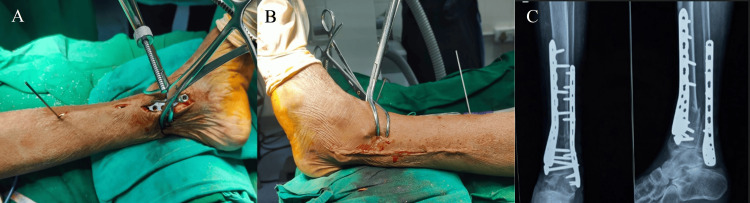
Intraoperative clinical and fluoroscopic images of MIPO tibial and ORIF fibular plating in closed distal tibial and fibular fractures (A) The technique of MIPO for the distal tibia involves indirect reduction using K wires and a reduction clamp. A sub-muscular plane is then created using a Cobb elevator, and a medial anatomical distal tibial locking plate is fixed. (B) The fibula is fixed using the standard ORIF technique. (C) Shows the postoperative radiograph. MIPO: minimally invasive plate osteosynthesis, ORIF: open reduction and internal fixation

Then, expose the tibia medially, proximal and distal to the fracture site, using the MIPO approach (Figure 3). Complete fracture reduction using indirect reduction methods such as pointed reduction forceps, manual traction, or bone spreaders. Use Cobb's elevator to create a submuscular tunnel. Pass the plate through the tunnel with a thread knotted to one end, tug it with a rongeur, and secure it with screws on either side under fluoroscopic guidance. Secure each fragment on either side by purchasing a minimum of six cortices.

If MIPO is not used due to a complex fracture pattern or inability to obtain indirect reduction, approach the tibia using the medial approach to the distal tibia, exposing as much as necessary. Hold the direct reduction of fracture fragments provisionally with K-wires, and place the plate in a buttress or bridging mode. Provide allogenic bone graft if needed due to excess metaphyseal comminution or bone gap. Check the reduction under fluoroscopic guidance, perform a wound wash, and close the wound in layers.

Postoperative protocol

All patients received a posterior above-knee pop slab, which was converted to a below-knee pop slab on the fourth postoperative day. The below-knee slab was kept for four to six weeks. Postoperative radiographs were taken on the second day after surgery. Patients were discharged from the hospital on the fifth postoperative day. The wound was examined on the second and fifth postoperative days, sterile dressing was applied, and sutures were removed on the 12th to 14th postoperative days. Static quadriceps, active knee bending, ankle ROM, and strengthening exercises began on the 14th postoperative day. Partial weight-bearing ambulation began at six weeks postoperatively, followed by complete weight-bearing at 12 weeks when sufficient callus was visible on a radiograph. Beginning around 12 weeks, all patients were able to bear full weight on the operated leg.

Collected data

During the surgery, the total duration of the procedure, total blood loss, and photographs of key steps (both clinical and fluoroscopic) were collected. Postoperative X-rays were taken on the second postoperative day. The start of weight-bearing and ankle ROM exercises was determined and initiated on a patient-by-patient basis, following an evaluation of the visual analog scale (VAS) pain score, surrounding edema, postoperative X-rays, and the rigidity and stability of fixation. Patients were followed up until they were fully weight-bearing and the soft tissue had healed. After six to eight weeks, partial weight-bearing was allowed, followed by complete weight-bearing after 12 weeks.

Data analysis

The data collected were entered into a Microsoft Excel spreadsheet (Microsoft Corporation, Redmond, WA, USA) and imported into SPSS Statistics version 26 (IBM Corp. Released 2019. IBM SPSS Statistics for Windows, Version 26.0. Armonk, NY: IBM Corp.), which was used for data analysis. The data were analyzed with the help of an expert from the Department of Community Medicine, IMS & SUM Hospital, Bhubaneswar, Odisha. Descriptive statistics were expressed as frequencies (percentages), means, medians, and standard deviations. Independent t-tests and Chi-square tests were performed where applicable. A p-value of 0.05 or less was considered statistically significant.

The patients were followed up regularly at the OPD at four weeks, three months, six months, 12 months, and 18 months after surgery. Telephonic follow-up was conducted if necessary. At each OPD visit, the patient was evaluated for the VAS pain score, Southampton wound healing score (for soft tissue healing), American Orthopaedic Foot and Ankle Society (AOFAS) hindfoot score (clinical outcome), and ankle ROM using a goniometer. The patients were also evaluated radiologically for signs of union, delayed union, and non-union.

## Results

**Figure 4:**
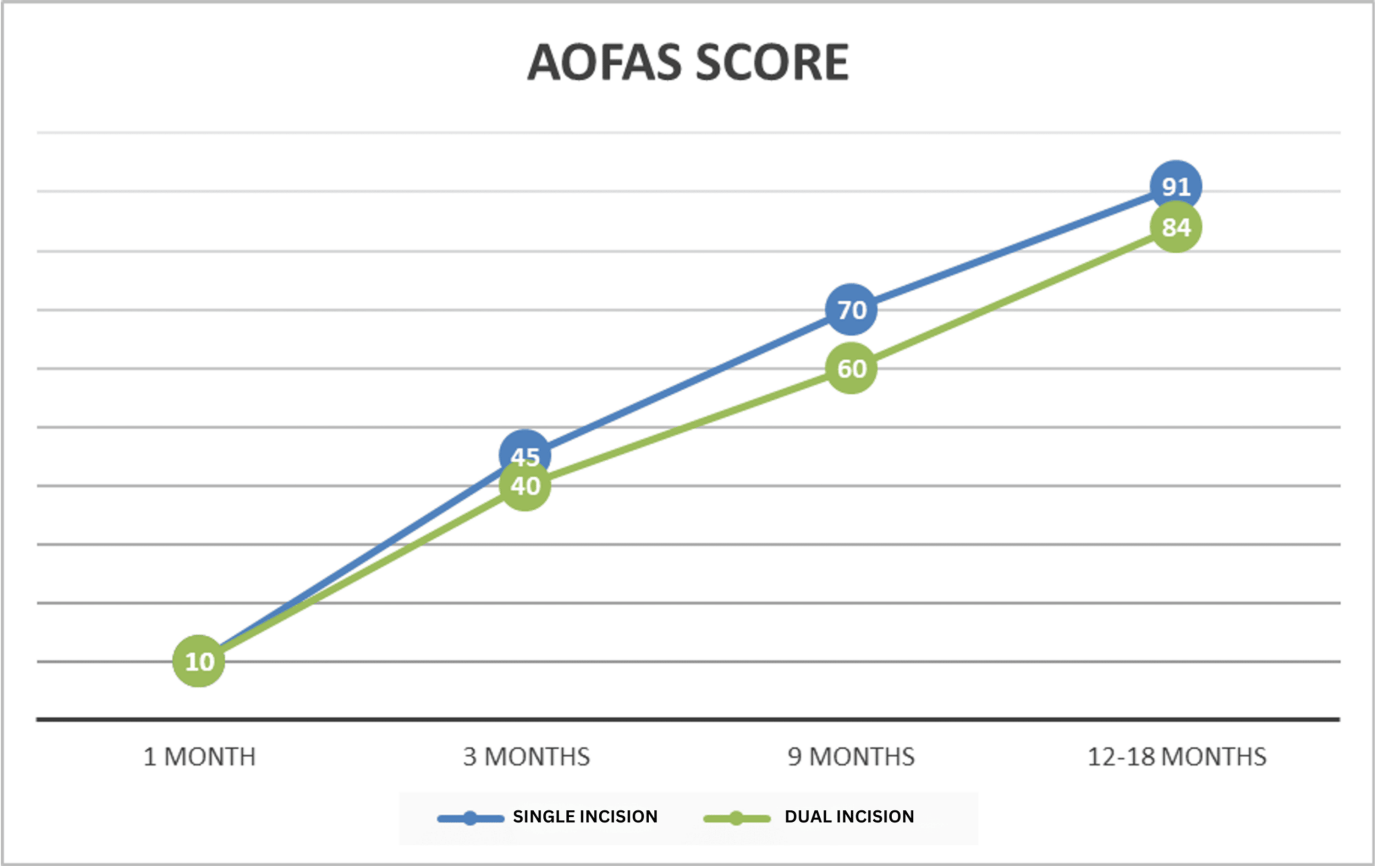
AOFAS score of single-incision and dual-incision groups AOFAS: American Orthopaedic Foot and Ankle Society

**Figure 5:**
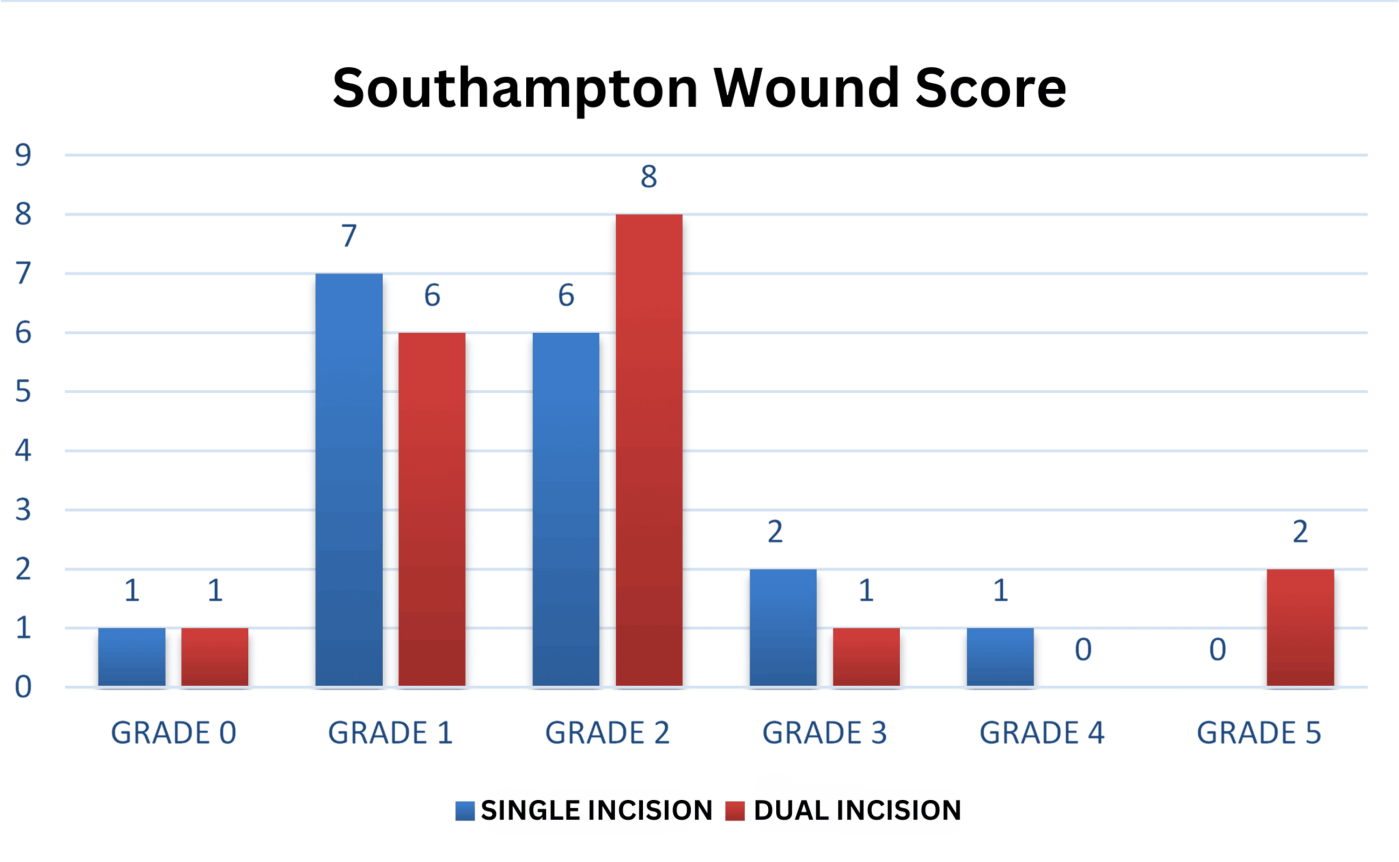
Southampton wound score of single-incision and dual-incision groups

We studied two groups of 40 skeletally mature patients (each group comprising 20 patients) with distal tibial and fibular fractures. Out of the 40 patients, three were lost to follow-up in the single-incision group and two in the dual-incision group. A total of 17 patients in the single-incision group and 18 patients in the dual-incision group were evaluated and compared. The mean follow-up duration was 12 months, and the average age of the patients was 50.3 years (Tables 1-2. Figures 4-5).

**Table 1:** Comparison of ankle ROM between the two groups at different time periods ROM: range of motion, SD: standard deviation, SE: standard error

		N	Mean	SD	SE	p-value
Ankle ROM postoperative (dorsiflexion)	Single incision	17	14.88	1.364	0.331	0.001
	Dual incision	18	11.44	2.502	0.59	
Ankle ROM at 12 months (plantarflexion)	Single incision	17	41.59	2.917	0.707	0.007
	Dual incision	18	33.56	2.684	0.633	

**Table 2:** Bivariate analysis * Independent t-test, NA: not applicable, VAS: visual analog scale, AOFAS: American Orthopaedic Foot and Ankle Society

Variables	Single incision	Dual incision	p-value
Age in years*	52.00 ± 14.87	48.78 ± 8.22	0.78
Operative time in minutes*	95.06 ± 7.04	109.89 ± 7.88	0.001
fracture to surgery (days)*	9.94 ± 1.46	10.94 ± 2.57	0.44
Blood loss (ml)*	202.41 ± 32.76	248.39 ± 28.18	0.001
Total duration of hospital stay*	9.65 ± 2.29	12.11 ± 3.12	0.001
Time to bony union*	21.41 ± 2.18	21.78 ± 2.51	0.67
VAS at postoperative*	8.00 ± 0.79	9.50 ± 0.51	0.023
VAS at 3 months*	5.41 ± 1.37	6.89 ± 0.90	0.001
VAS at 6 months*	3.12 ± 1.11	5.06 ± 0.87	0.001
VAS at 12 months*	1.35 ± 0.49	2.33 ± 1.32	0.001
AOFAS at postoperative*	11.24 ± 2.90	10.39 ± 2.20	0.006
AOFAS at 3 months*	43.76 ± 2.77	39.89 ± 3.35	0.005
AOFAS at 6 months*	69.88 ± 3.83	60.06 ± 3.24	0.030
AOFAS at 12 months*	91.47 ± 2.55	83.33 ± 8.71	0.001
Ankle dorsiflexion*	(15+/-2 degrees)	(11+/-3 degrees)	0.001
Ankle plantarflexion*	(41+/-3 degrees)	(35+/-4 degrees)	0.001
Wound complications	0	4	NA
Malunion/nonunion	0	1	NA
Delayed union	1	3	NA
Ankle stiffness	3	4	NA
Wound dehiscence	0	2	NA

## Discussion

In the present study, patients were aged from 22 to 73 years. The most common age group was 51-60 years. The mean age was 50.34 years, with a standard deviation of 11.85 years. There were 24 male patients and 11 female patients, resulting in a male-to-female ratio of 2.2:1. Fracture classification revealed 15% as 43-C (complete articular), 5% as 43-B (partial articular), and 80% as 43-A (extra-articular) according to the Muller AO classification. Most of the patients (68.6%) had experienced road traffic accidents, followed by falls from height (17.1%), slips and falls (8.6%), and falls from stairs (5.8%). Around 57.1% of patients had left-side injuries, while the remaining 42.9% had right-side injuries. Five patients had diabetes, three were smokers, and four patients had idiopathic hypertension.

The mean operative time was 102.6 ± 10.53 minutes. The mean blood loss was 226 ± 38.02 ml. The mean time to bony union was 21.60 ± 2.32 weeks. Likewise, the mean duration of hospital stay was 10.91 days, with a standard deviation of 2.98 days. The time to bony union did not differ statistically in the comparison, and all patients achieved bony union at an average of 21.60 weeks with a standard deviation of 2.329 weeks.

In the study by Richard et al. [13], it was suggested that there is a higher risk of medial hardware exposure and infection in MIPO of distal tibial fractures due to its subcutaneous placement. Additionally, it can disturb day-to-day activities and cause discomfort to the patient due to implant prominence.

In our study, we examined the soft tissue condition of patients from admission to postoperative follow-ups. The wound condition was assessed at admission for blisters, bruises, and local swelling. The skin condition was evaluated every 24 hours, and once wrinkling signs appeared or adequate reduction in swelling was achieved, the patients were planned for surgery.

The mean operative time was significantly shorter in the single-incision group (95.06 ± 7.04 minutes) compared to the dual-incision group (109.89 ± 7.88 minutes) (p<0.05). The mean blood loss was lower in the single-incision group compared to the dual-incision group (p<0.05), and the total duration of hospital stay was also shorter in the single-incision group (p<0.05) (Table 2). No nerve injuries were observed in either group. Similar results were reported by Lin et al., who found that the single-incision approach was associated with fewer soft tissue complications. However, their study noted an increased risk of nerve injuries, particularly superficial peroneal nerve injuries, as it is prone to injury during exposure [14].

In the study by Zhang et al., it was suggested that bone union was better in the single-incision group (p<0.05) when they conducted a retrospective study comparing single-incision and dual-incision plating in the mid-to-lower segment of tibial and fibular fractures among 212 patients [15]. In contrast, this study found no statistically significant difference in the time to bony union between the groups; all patients achieved bony union at an average of 21.60 weeks with a standard deviation of 2.329 weeks.

The delay in ORIF was not significantly different (p>0.05) between the single-incision and dual-incision groups. However, it was observed that the lateral approach could be performed once the lateral aspect of the ankle was suitable for surgery, whereas the dual-incision approach required an average of one to two more days for swelling resolution. Femino and Vaseenon described that the single-incision approach could reduce the need to delay ORIF in pilon fractures [16].

The study conducted by Lin et al. suggested similar results, i.e., fewer soft tissue complications were observed in the single-incision group compared to the dual-incision group [14]. The study by Shantharam et al. [17] also suggested that the single-incision approach causes less trauma to soft tissues, leading to better wound healing. In the single-incision group, wound healing was observed in seven out of 17 patients after erythema and inflammation (redness, swelling, hemoserous discharge), with eventual excess slough and scar formation, resulting in Southampton wound scores of 2C, 2D, and 3A. One patient had an infection with a wound grade of 4A, which eventually healed with culture-guided antibiotics and dressing. The remaining nine patients healed with minimal inflammation, with Southampton scores of 1B and 2A (Figure 4). All wounds eventually healed with regular dressing, and no patient experienced wound dehiscence or hardware exposure. One patient had a preoperative extensor tendon injury, which was repaired intraoperatively.

In the dual-incision group, soft tissue healing showed mild erythema and inflammation signs in seven patients (Southampton wound scores of 1C, 2B, and 2C) and excess erythema, inflammation, and slough in nine patients (scores of 2A, 3A, and 3B), all of which healed eventually with regular dressing.

**Figure 6:**
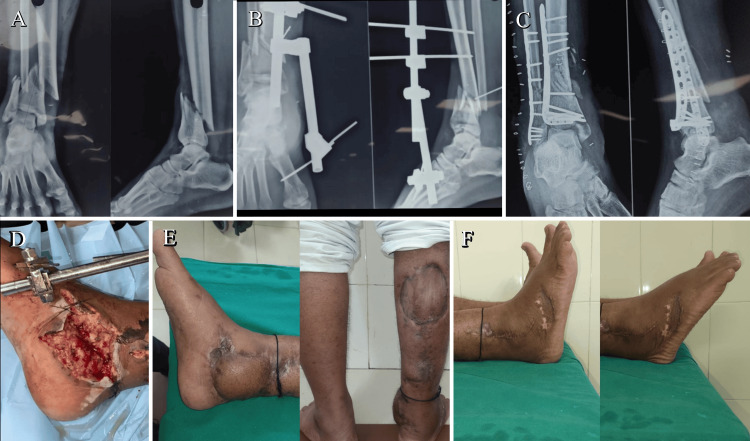
Case 1 single-incision approach (A) A 55-year-old male patient presented with a Gustilo-Anderson compound Grade 3B extra-articular fracture of the distal tibia and fibula. (B) The patient was managed with external fixation and underwent plastic surgery for appropriate wound management. (C) After soft tissue healing, the patient was scheduled for a single-incision direct lateral approach for distal tibial and fibular plating. (D) The medial compound wound was assessed at the time of external fixation. (E) A 10-month follow-up of the same patient showed good soft tissue healing and pain-free weight-bearing. (F) The patient demonstrated good ankle ROM, with 15 degrees dorsiflexion and 35 degrees plantarflexion. ROM: range of motion

**Figure 7:**
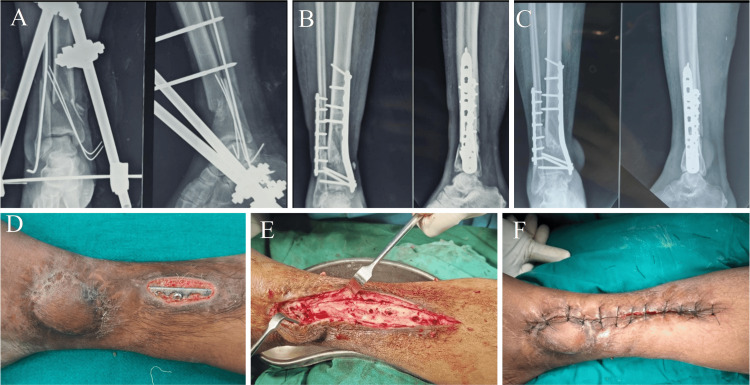
Case 2 dual-incision approach (A) A 40-year-old female with a history of RTA resulting in an open distal tibial and fibular fracture, Grade 3b. The compound wound was on the medial side and was managed with external fixation. (B) After plastic surgery intervention, the patient was treated with MIPO tibia and ORIF fibula. (C) X-ray after 11 months of follow-up. (D) Follow-up showed wound dehiscence and implant exposure on the medial side. (E, F) Implant removal was performed after 11 months. RTA: road traffic accident, MIPO: minimally invasive plate osteosynthesis, ORIF: open reduction and internal fixation

**Figure 8:**
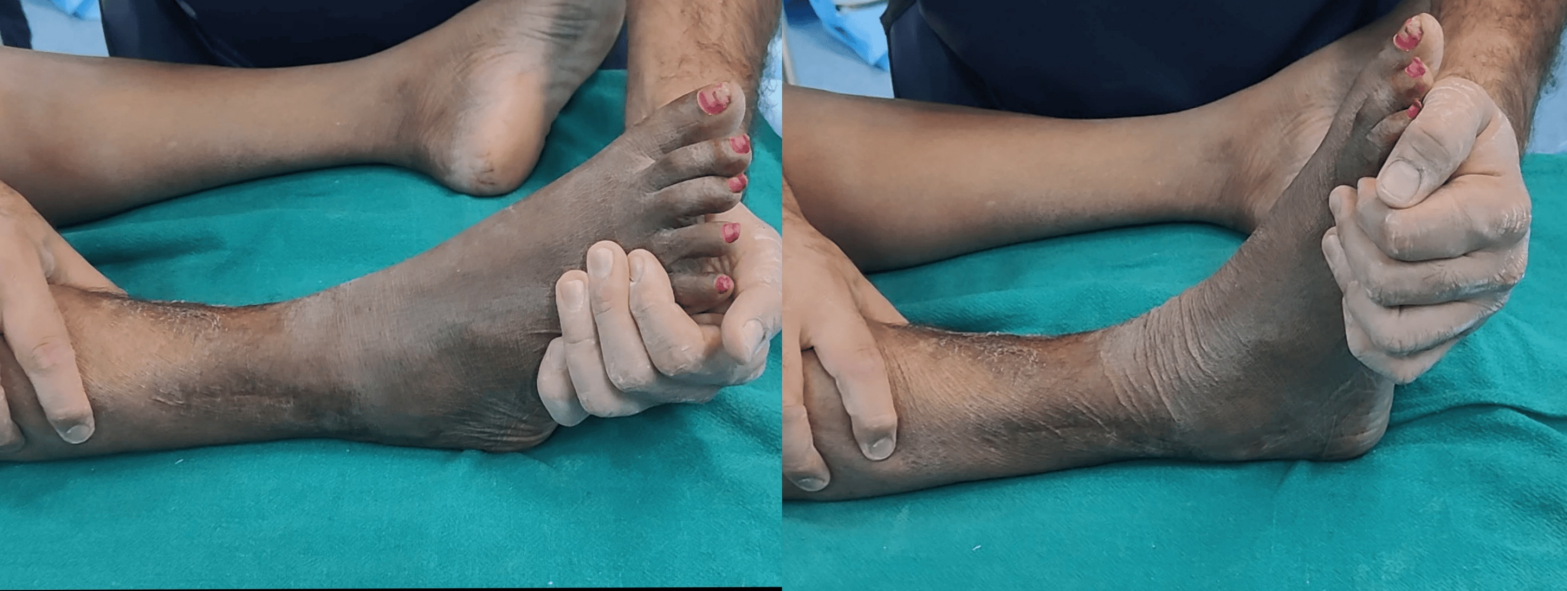
Restricted ankle ROM in the dual-incision approach This is the passive ankle ROM of the case in Figure 7 (MIPO tibia and ORIF fibula) after a follow-up of 11 months. The ankle plantarflexion (30 degrees) and dorsiflexion (10 degrees) are restricted. ROM: range of motion, MIPO: minimally invasive plate osteosynthesis, ORIF: open reduction and internal fixation

**Figure 9:**
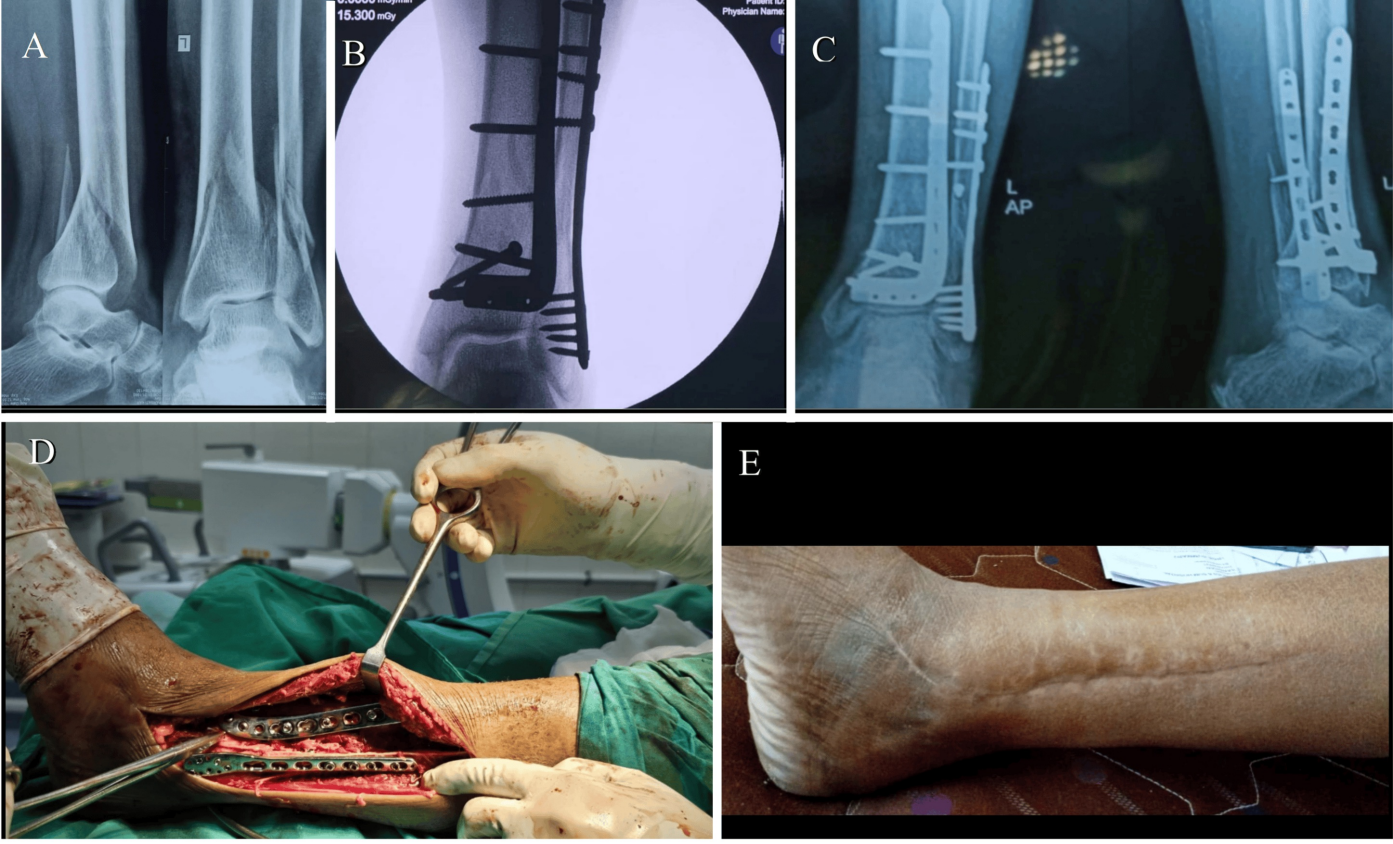
Case 3 single-incision approach (A) A 50-year-old male with a closed distal tibial and fibular fracture due to a road traffic accident (RTA). (B, D) Intraoperative fluoroscopic and clinical images as the case was managed using a single-incision direct lateral approach. (C, E) 12-month follow-up of the patient showing good radiological outcomes and good soft tissue healing.

**Figure 10:**
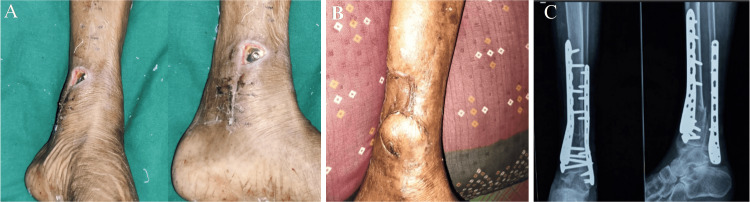
Case 4 dual-incision approach (A) A 53-year-old female was managed with a dual-incision approach (MIPO tibia and ORIF fibula), showing deep skin necrosis and implant exposure. (B) She was treated with flap coverage by plastic surgery, which eventually healed. (C) Radiograph taken two months postoperatively. MIPO: minimally invasive plate osteosynthesis, ORIF: open reduction and internal fixation

When the medial side of the ankle had a compound wound that required plastic surgery intervention, the external fixator was removed once the wounds had healed, and the patient was planned for definitive fixation with plates and screws, either through a direct lateral approach or a dual-incision approach. In such cases, it was observed that the single-incision direct lateral approach was a better option, as it avoided trauma to the flap cover provided during the plastic surgery intervention (Figures 6-10).

A 50-year-old female patient who had a compound wound on the medial side, which was treated with an external fixator followed by a flap cover from plastic surgery, underwent ORIF plating using a dual-incision approach (MIPO tibia, ORIF fibula). She encountered an infection of the flap cover wound and, at the 12-month follow-up, showed a Southampton wound score of 5 (pus discharge with soft tissue breakdown and implant exposure), which was managed with implant removal and wound debridement (Figure 6).

Another case of a compound distal tibia and fibula, which was managed with external fixation followed by a flap cover on the medial aspect of the ankle, was planned for a single-incision direct lateral approach. This showed much better soft tissue healing, lower rates of infection, earlier bone union, and better ankle ROM compared to the dual-incision approach. A total of two out of 18 patients in the dual-incision group experienced wound breakdown, which required plastic surgery intervention (Figure 5).

The AOFAS hindfoot score was not significantly different at one and three months follow-up (p>0.005) but was higher in the single-incision approach patients at six and 12 months follow-up (p<0.05), as depicted in Table 2. At 12-month follow-up, the single-incision approach patients had better overall ankle function and fewer complaints of pain and swelling during weight-bearing activities. In the dual-incision group, eight out of 18 patients complained of edema and swelling in the ankle and foot region after prolonged standing, walking, or working. No such complaints were reported in the single-incision group. The return to recreational and regular activities was better in the single-incision group, while the dual-incision group reported that most patients experienced limitations in recreational activities, such as brisk walking, with mild limitations in daily activities like walking, squatting, and prolonged standing. The patients in the single-incision group had better functional outcomes when walking on uneven surfaces and climbing stairs. All these parameters were measured using the AOFAS ankle-hindfoot score system.

The VAS pain score was consistently lower in the single-incision patients at three, six, and 12 months of follow-up (p<0.05) across all periodic assessments. Postoperative ankle ROM was consistently better in the single-incision group, with an average ROM of 41 ± 3 degrees for plantar flexion and 15 ± 2 degrees for dorsiflexion in 16 out of 17 cases. One case of ankle stiffness, due to a compound wound, was observed (p<0.05). In the dual-incision group, the average ankle ROM was 10 ± 3 degrees for dorsiflexion and 35 ± 4 degrees for plantar flexion in 14 out of 18 cases, with good ankle ROM (15/40 degrees) in two cases. In the study by Lin et al., different results were observed, as they suggested no difference in overall ankle ROM between the two comparison groups [14].

The highlight of this study is the comparison of wound healing grades using the Southampton wound healing score and functional outcomes using the AOFAS hindfoot score between the single-incision and dual-incision groups, which had not been conducted by previous authors in the literature.

The dual-incision approach to the distal tibia and fibula is commonly used due to its sufficient exposure and direct manipulation. The single-incision approach could be utilized in fractures of the distal third of the tibia and fibula, particularly in AO types A1, A2, B1, and C1 distal tibial fractures, as well as type C1 fibular fractures, according to the AO classification system.

The preoperative planning for fracture fixation included obtaining a CT scan, which revealed that a single-incision approach was indicated for patients with a fracture fragment in the anterolateral plafond of the tibia. However, if the fracture fragment was far medial, a dual-incision approach was recommended to access the fragment directly, reduce it, and apply a buttress plate for fixation. Similar findings were suggested in the study by Femino et al. [16], where a dual-incision approach was used when necessary to access the medial side of the fracture, either openly or percutaneously. The study by Shantharam et al. [17] also recommended a single-incision approach to reach the lateral plafond of the tibial articular surface, which could not be accessed via a medial approach to the distal tibia. The single-incision approach is a useful surgical technique for managing distal tibial fractures in cases where there is no medial comminution of the tibia and when the fibula needs to be fixed [18].

The anatomical basis of the anterolateral approach revealed a clear angiosome interval (anterior tibial artery and anterior peroneal artery), intermuscular plane, and nerve interface in the lateral lower leg, as first described by Taylor et al. in their study, which detailed the anatomical basis of the angiosomes of the leg and ankle and the placement of incisions between the angiosomes to preserve the skin's blood supply. The anterior perforating peroneal artery emerges from the deep posterior compartment and travels via the anterior hiatus between the interosseous membrane and the proximal side of the syndesmosis [11].

The superficial and deep peroneal nerves, along with the anterior tibial arteries, were clearly exposed and protected, reducing the risk of neurovascular injury. However, the potential for nerve injury and challenges related to the removal of internal fixation requires careful consideration. The single-incision approach demonstrated fewer soft tissue complications, improved ankle ROM, lower pain scores, and earlier return to activities, consistent with the initial hypothesis. Given the study's limitations, including a small sample size and short-term follow-up, further research with larger cohorts and long-term evaluations is necessary to confirm the safety and efficacy of single-incision approaches for distal tibial and fibular fractures.

## Conclusions

The study compared the efficacy of the single-incision direct lateral approach and the MIPO/dual-incision approach in surgically managing distal tibial and fibular fractures. The direct lateral approach demonstrated a potential advantage in preserving soft tissue integrity and achieving better functional outcomes for the ankle, which is important for reducing the risk of complications such as infection and ankle stiffness and promoting faster healing.

The single-incision approach presents itself as a promising alternative for managing distal tibial and fibular fractures, primarily due to its potential for better soft tissue preservation. By minimizing trauma to the surrounding soft tissues, this approach may contribute to improved outcomes and faster recovery for patients undergoing surgical intervention for these types of fractures.

While both single-incision and dual-incision approaches yield comparable clinical outcomes, the former offers the advantage of enhanced soft tissue conservation and better functional outcomes. However, surgeons must exercise caution, particularly in mitigating the risk of nerve injury, especially during the initial learning phases. Despite these challenges, the single-incision approach represents a viable option for effectively managing distal tibial and fibular fractures, potentially leading to better patient outcomes and recovery.
